# Free-Standing Sodium Titanate Ultralong Nanotube Membrane with Oil-Water Separation, Self-Cleaning, and Photocatalysis Properties

**DOI:** 10.1186/s11671-020-3255-9

**Published:** 2020-01-28

**Authors:** Shuling Shen, Cheng Wang, Minquan Sun, Mengmeng Jia, Zhihong Tang, Junhe Yang

**Affiliations:** School of Materials Science and Engineering, University of Shanghai for Science and Technology, Shanghai, 200093 People’s Republic of China

**Keywords:** Free-standing, Sodium titanate ultralong nanotubes, Oil-water separation, Self-cleaning, Photocatalysis

## Abstract

In this work, a free-standing sodium titanate ultralong nanotube membrane for multifunctional water purification has been prepared. For obtaining this free-standing membrane with good tenacity, one-dimensional (1D) sodium titanate ultralong nanotubes with a diameter of about 48 nm and length of hundreds of micrometers were prepared from TiO_2_ nanoparticles by a stirring hydrothermal method, which can be easily assembled into 2D membranes by facile vacuum filtration. After modified with methyltrimethoxysilane (MTMS), the free-standing membrane with hydrophobic surface possesses oil-water separation, self-cleaning and photocatalytic functions at the same time, which is favorable for the recovery of membrane and decontamination of various pollutants including oils, dust, and organic dyes from water. Furthermore, this membrane also exhibits excellent alkaline, acid, and corrosive salt resistance. This free-standing sodium titanate membrane with multifunction has potential applications in efficient wastewater purification and environmental remediation.

## Introduction

Oily water, arising from industrial sewage and frequent oil spill accidents, is harmful to the environment, animals, plants, and even humans and has aroused widespread concern throughout the world. The removal of intractable oil from water is a tough job [1, 2]. At present, many treatment methods for oily wastewater have been developed. Membrane separation technology has attracted much attention for its advantages of low energy consumption, flexibility, environmental friendliness, and high single-stage separation efficiency [3, 4]. Many researches have been carried out on improving the sustainability and efficiency of membrane separation technology. Szekely et al. noted that a large amount of wastewater generates during the fabrication process of the polymeric membrane, which makes membrane separation technology not as green as it is known. For making the membrane technology greener and more sustainable, they proposed a continuous wastewater treatment process to remove over 99% of the organic impurities by adsorbents and reuse these purified waters for the fabrication of membrane without detrimental effects on the performance of the final membrane [5]. They also revealed the direct and indirect effects of the polarity of treatment solvent on membrane performance through systematical studies, which was successfully applied for improving the efficiency of pharmaceutical purification [6]. More recently, many nano-engineering techniques were developed for the precise fabrication of porous membranes to meet specific separation required. Yang et al. prepared a solvent-free crystallization of MOF (ZIF-8) membranes by a layer-by-layer deposition process. The defect-free ZIF-8 membrane exhibited both higher H_2_ permeability and higher H_2_/CO_2_ selectivity simultaneously than the ever reported ZIF-8 membranes [7]. Inspired by marine mussel, Szekely et al. for the first time fabricated a nanoengineered membrane formed by in situ polymerization of dopamine within a PBI support for the separation of polar aprotic solvents. The coating of PDA eliminated covalent cross-linking of the PBI backbone and achieved the highest permeance value of DMF [8]. Manufacturing membranes with functional materials endow the membrane multifunction besides separation. Xu et al. reported a composite membrane composed of LiNbO_3_ coating layer and poly(ether sulfone) (PES) support. The presence of LiNbO_3_ endowed the membrane photocatalytic denitrification function [9]. Multifunction membranes are aspired to effectively remove oil from various wastewaters [10–12].

Recently, more and more 1D inorganic materials were applied for obtaining free-standing membrane owing to their large specific surface area, low density, and high thermal conductivity and chemical sensitivity, as well as tunable metal and semiconductor properties [13–16]. 1D titanate materials not only have unique layered structure, good electrochemical, and optical properties but also possess excellent mechanical properties. These characteristics make it possible to be used in the fields of photocatalysis [17], adsorption [18, 19], sodium-ion battery [20], and energy storage [21]. Recently, Wang et al. prepared a membrane for high-efficiency oil/water emulsion separation by using sodium titanate nanofibers, which were supported on a cellulose microfiber layer [22]. In this work, a free-standing membrane was prepared by only using sodium titanate ultralong nanotubes with a length of hundreds of micrometers. This free-standing membrane exhibited excellent flexibility. After being modified with methyltrimethoxysilane (MTMS), the free-standing hydrophobic membrane possessed oil-water separation, self-cleaning, and photocatalysis functions, which are favorable for the recycling of separation membranes.

## Methods

### Materials

TiO_2_ powder (P25) was purchased from Deguassa Co. Ltd, Germany. Methyltrimethoxysilane (MTMS, ≥ 98%) and ethanol (CH_3_CH_2_OH, ≥ 95%) were purchased from Aladdin Reagent Company, China. Hydrochloric acid (HCl, 37%), sodium hydroxide (NaOH, ≥ 96%), and oxalic acid (≥ 99.5%) were obtained from Sinopharm Chemical Reagent Co. Ltd. All the chemical reagents were used in the experiment process without further purification. The deionized (DI) water was used throughout this experiment.

### Synthesis of Na_2_Ti_3_O_7_ Ultralong Nanotubes

The synthesis of Na_2_Ti_3_O_7_ ultralong nanotubes was according to the literature procedure [22, 23]. Typically, 0.2 g of P25 powder was added to 30 mL of 10 M NaOH aqueous solution with continuous stirring for 5 min. Then the slurry was transferred into 50 mL Teflon-lined stainless-steel autoclave with a magnetic stirrer. The autoclave was put inside a silicon oil bath and the reaction temperature was set at 130 °C for 24 h. The stirring speed is 300 rpm. After the reaction, the autoclave was cooled to room temperature naturally. The precipitate was recovered and washed with distilled water several times to remove excess NaOH. The obtained product was further cleaned by using 0.1 M HCl solution three times to produce high purity Na_2_Ti_3_O_7_ ultralong nanotubes and washed again with distilled water for several times until pH = 7.

### Synthesis of Free-Standing Na_2_Ti_3_O_7_ Porous Membrane and Surface Modification

Free-standing Na_2_Ti_3_O_7_ porous membrane was prepared by simple vacuum filtration without any other additives. Typically, Na_2_Ti_3_O_7_ ultralong nanotubes dispersing in ethanol with different concentrations were poured into the filter bottle and vacuum filtered for 10 min. The obtained membrane was dried in room temperature. By using different amounts of Na_2_Ti_3_O_7_ ultralong nanotubes, porous membranes with weights of 30 mg, 45 mg, 60 mg, and 75 mg were obtained, which are correspondingly defined as F-30, F-45, F-60, and F-75.

The obtained membranes were modified by dipping in MTMS sol-gel solution for 30 s and dried in room temperature for one night.

### Characterization

The morphology and size of the obtained samples were examined on a Tecnai G2 F30 S-Twin transmission electron microscope (TEM, FEI, USA) operated at 200 kV. The morphologies of the membranes were characterized by using a field emission scanning electron microscope (SEM, Hitachi S4800). Powder X-ray diffraction (XRD) patterns were recorded on a Bruker D8 Advance powder X-ray diffractometer at a scanning rate of 4° min^−1^, with Cu-Kα radiation (*λ* = 1.5406 Å) in the range of 10–60°. The contact angle (CA) of the membranes was measured on a Krüss DSA 30 (Krüss Company, Ltd., Germany) apparatus.

## Results and Discussion

### Na_2_Ti_3_O_7_ Ultralong Nanotubes and Free-Standing Membrane

**Fig. 1:**
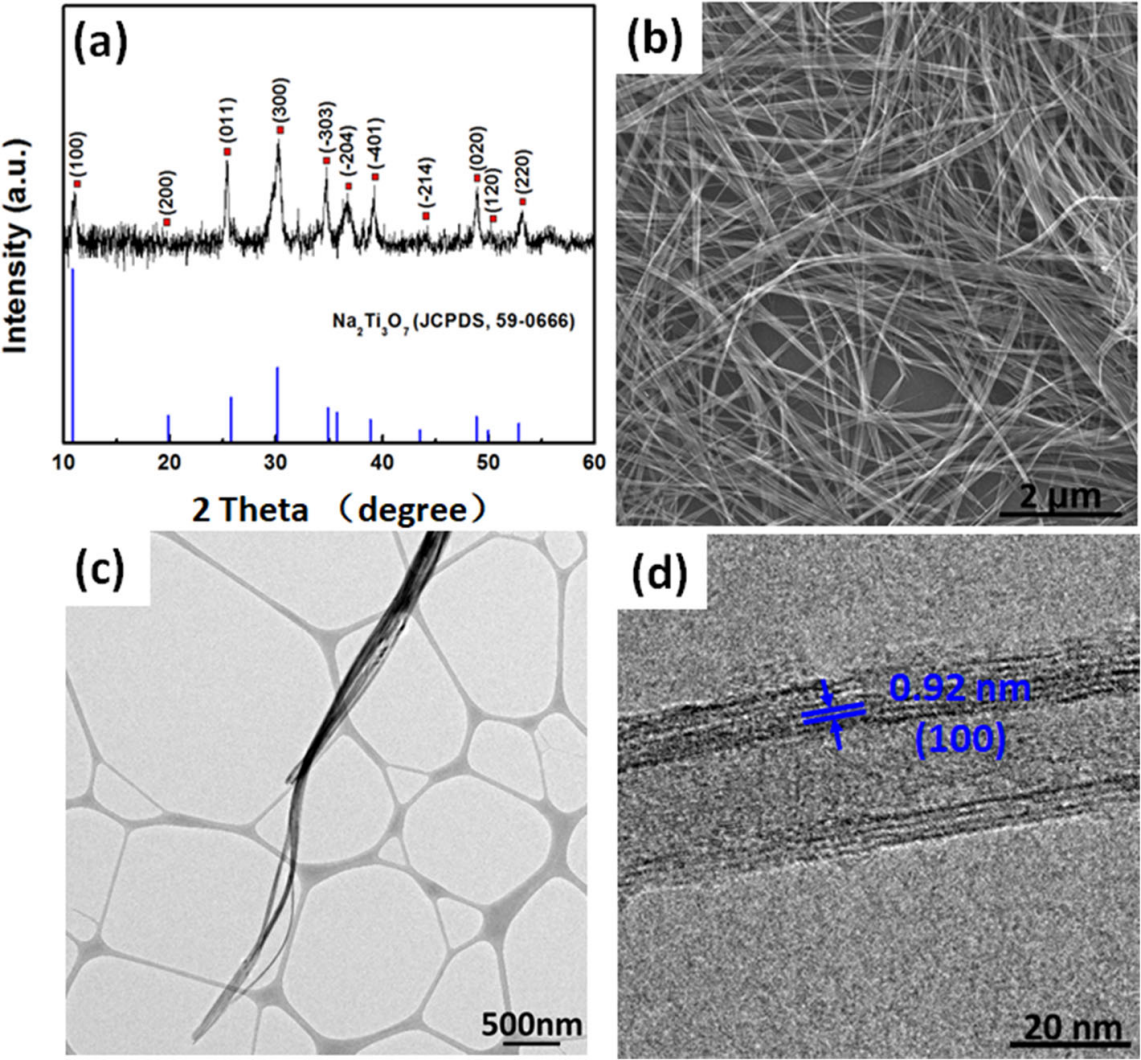
**a** XRD pattern, **b** SEM, **c** TEM, and **d** HRTEM of Na_2_Ti_3_O_7_ ultralong nanotubes

Figure 1a is the XRD patterns of the product synthesized by stirring hydrothermal method. It can be seen that there are characteristic peaks at 11.1°, 18.8°, 25.4°, 30.3°, 34.8°, 36.7°, 39.2°, 44.2°, 48.9°, 50.2°, and 53.1°, which can be indexed as (100), (200), (011), (300), (− 303), (− 204), (− 401), (− 214), (020), (120) and (220) planes of Na_2_Ti_3_O_7_ (JCPDS, 59-0666), respectively [24, 25]. The basic building block of this kind of sodium titanate structure is TiO_6_ octahedron, the edge of which forms a negatively charged layered structure, and the opposite cation of Na^+^ is located between adjacent layers, resulting in a variable layer spacing [26–28]. XPS measurement further confirms the presence of Na, Ti, and O in the product with an atomic ratio of 1:1.58:4.04, which is in respect to the composition of Na_2_Ti_3_O_7_ (Additional file 1: Figure S1). Figure 1b shows the SEM image of the obtained Na_2_Ti_3_O_7_, which looks like ultralong “nanobelts”. It can be seen that the length of Na_2_Ti_3_O_7_ “nanobelts” can reach up to hundreds of micrometers with good flexibility, which will favor the formation of free-standing porous membranes. The ultralong “nanobelts” with excellent flexibility tend to array along the axis (Fig. 1c). However, high-resolution transmission electron microscope (HRTEM) image of a typical single “nanobelt” indicates that the “nanobelt” is actually a nanotubular structure (Fig. 1d). The lattice distance of 0.92 nm is corresponding to the interlayer spacing of (100) facet of layered Na_2_Ti_3_O_7_, suggesting the multiwall nanotubular structure of Na_2_Ti_3_O_7_.

**Fig. 2:**
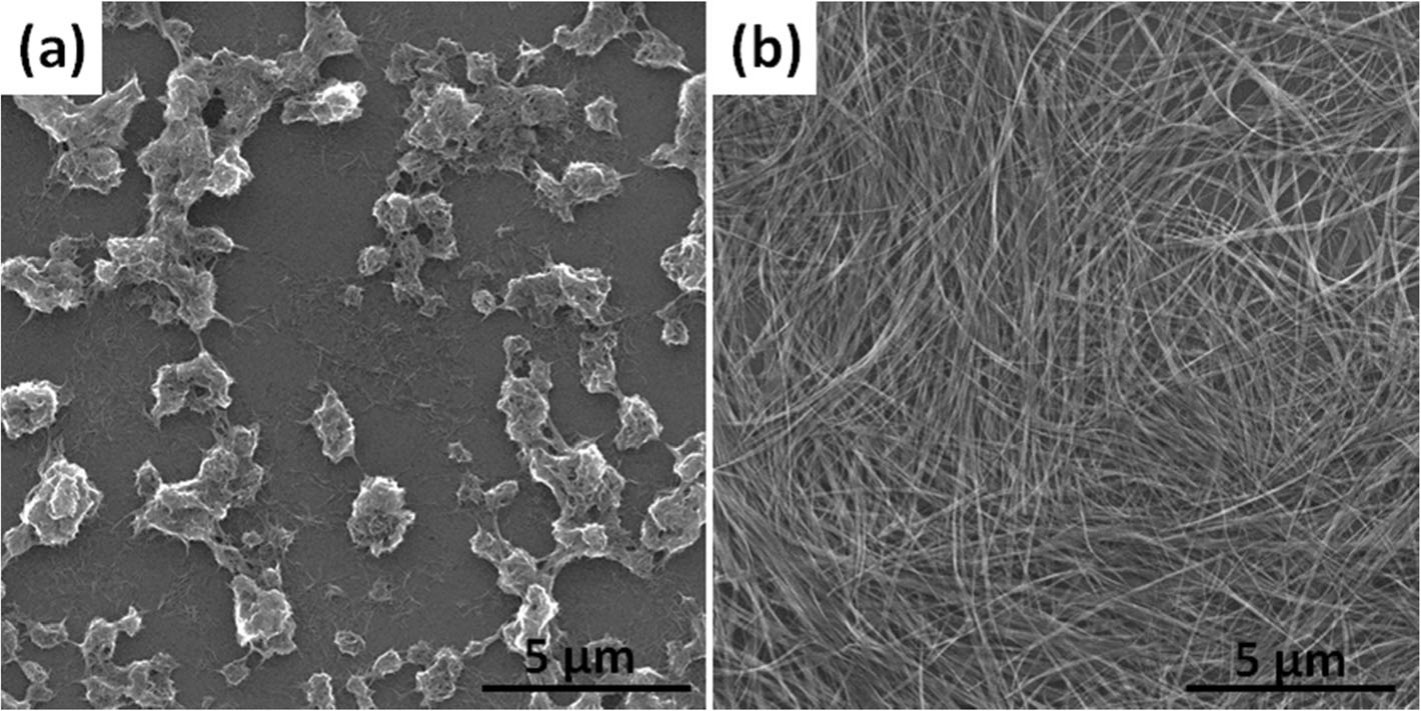
SEM images of Na_2_Ti_3_O_7_ nanotubes synthesized by hydrothermal method with 0 rpm (**a**) and 300 rpm (**b**)

**Fig. 3:**
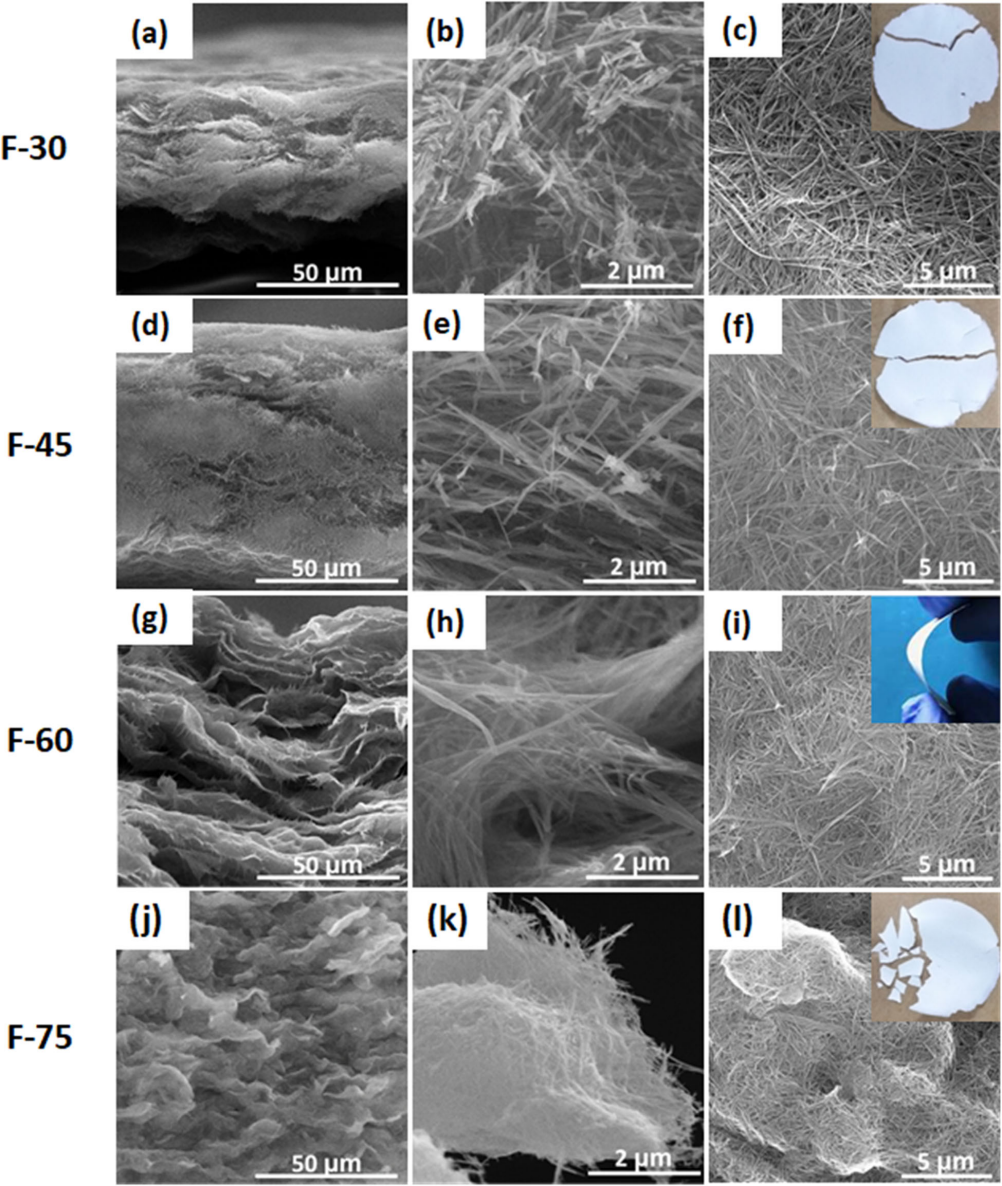
SEM images of cross-section of F-30 (**a**, **b**), F-45 (**d**, **e**), F-60 (**g**, **h**), and F-75 (**j**, **k**). TEM images of top view of F-30 (**c**), F-45 (**f**), F-60 (**i**), and F-75 (**l**). The insets are the optical images of corresponding membranes

**Fig. 4:**
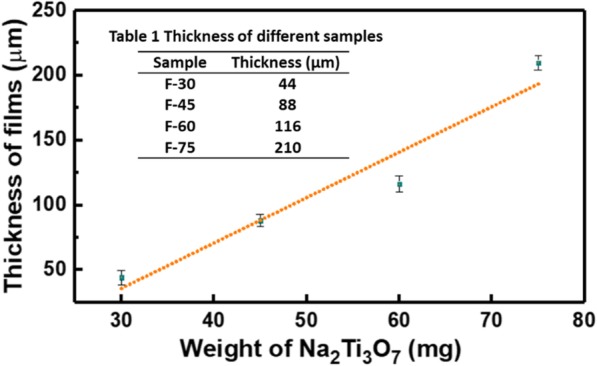
The plot of thickness versus the weight of the membrane

In this study, the Na_2_Ti_3_O_7_ ultralong nanotubes were synthesized by hydrothermal method with stirring. Sun et al. [29] have systemically studied the formation mechanism of Na_2_Ti_3_O_7_ nanotubes in hydrothermal process without stirring. Generally, the length of Na_2_Ti_3_O_7_ nanotubes synthesized in the hydrothermal process without stirring is about 500 nm. These short nanotubes easily aggregate, which is not conducive to the formation of membranes (Fig. 2a). It has been reported that the length of titanate nanotubes can be controlled by a rotation speed during hydrothermal reaction [23, 30]. We found that the elongated Na_2_Ti_3_O_7_ nanotubes are easy to lay flat to form a film (Fig. 2b). But if using these Na_2_Ti_3_O_7_ nanotubes to form a free-standing membrane, polymer supports such as polyethylenimine (PEI) must be used [31]. For obtaining a free-standing membrane without polymer supports, the amount of Na_2_Ti_3_O_7_ ultralong nanotubes was studied. SEM and TEM images in Fig. 3 indicate that the membranes consist of randomly oriented ultralong nanotubes and with the increase of membrane weight, Na_2_Ti_3_O_7_ ultralong nanotubes are denser. Figure 3a–f indicates that when the amount of Na_2_Ti_3_O_7_ ultralong nanotubes is small (30 mg and 45 mg), the assembly of Na_2_Ti_3_O_7_ ultralong nanotubes is loose and the adhesion between the nanotubes is insufficient. So, although these membranes have a certain tenacity but they tend to split into halves when they are bent (insets in Fig. 3c and f). But when the weight of membrane reaches up to 75 mg, this high content of nanotubes heavily intertwine, which leads to less freedom interspace between nanotubes and uneven of the membrane (Fig. 3j–l). Consequently, F-75 membrane with less tenacity is easily broken into small pieces (inset in Fig. 3l). F-60 membrane displays excellent tenacity due to its moderate nanotubes content, relative freedom between each other, and sufficient adhesion (Fig. 3g–i). So, F-60 was used for further studies. Additional file 1: Figure S2a–d indicates the corresponding thicknesses of F-30, F-45, F-60, and F-75 are 44 μm, 88 μm, 116 μm, and 210 μm, respectively (Table 1, Fig. 4). The thicknesses of these membranes have a linear relationship with the weight of Na_2_Ti_3_O_7_ ultralong nanotubes (Fig. 4). These results suggest that the thickness and tenacity of the membranes can be tuned through controlling the amount of Na_2_Ti_3_O_7_ ultralong nanotubes.

**Table 1:**
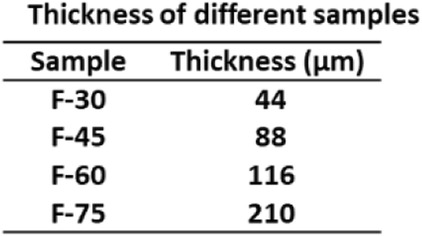
Thickness of different samples

### Wettability of the F-60 Membrane

**Fig. 5:**
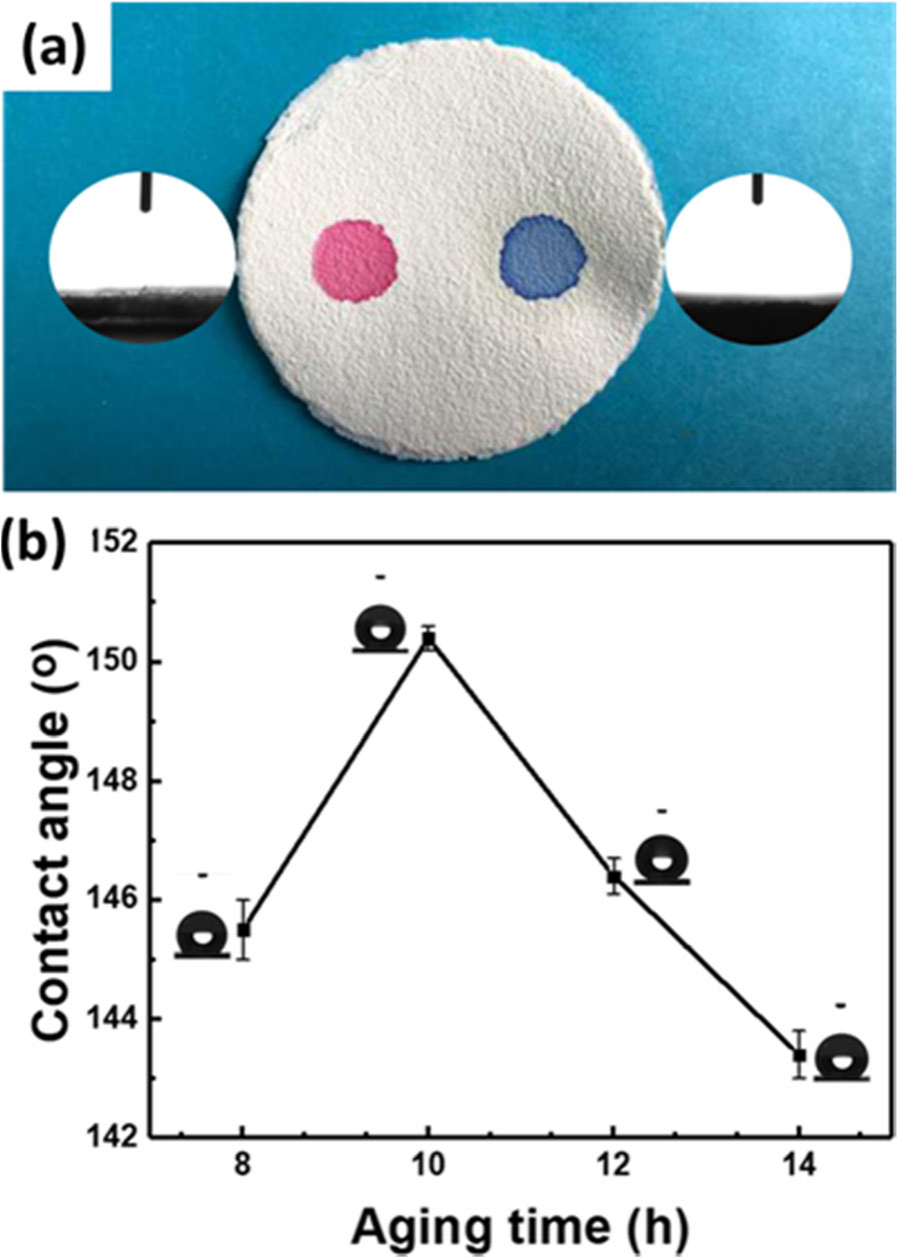
**a** Optical photo of the F-60 membrane dropped with carbon tetrachloride (left side, stained by methyl red) and water (right side, stained by methylene blue). **b** Effect of aging time of MTMS on the contact angle of the modified F-60 membrane

Figure 5a indicates that both carbon tetrachloride (left side, stained by methyl red) and water (right side, stained by methylene blue) can spread and permeate the obtained F-60 membrane. The surface tension of carbon tetrachloride and water is 26.1 mN m^−1^ and 72.8 mN m^−1^ [32], respectively. In order to obtain a hydrophobic membrane for separating oil-water mixture, the surface tension of the F-60 membrane must be lower than ¼ of pure water (about 18 mN m^−1^) [33]. Then the obtained F-60 membrane must be modified. In our study, the free-standing F-60 membrane is easily modified by dipping in MTMS sol due to its low surface energy and micro-nano rough structure [34–36]. The aging time of MTMS sol has an effect on the contact angle of the modified F-60 membrane. Figure 5b displays that with the increase of aging time, the contact angle of the modified F-60 membrane increase. But when the aging time is 14 h, the contact angle decreases. Because with the increase of aging time, MTMS gel with poor fluidity forms, which leads to the uneven surface of the F-60 membrane (Additional file 1: Figure S3) and the decrease of contact angle [37]. The aging times range between 10 and 12 h are suitable for obtaining a hydrophobic membrane.

### Multifunction of the Modified F-60 Membrane

**Fig. 6:**
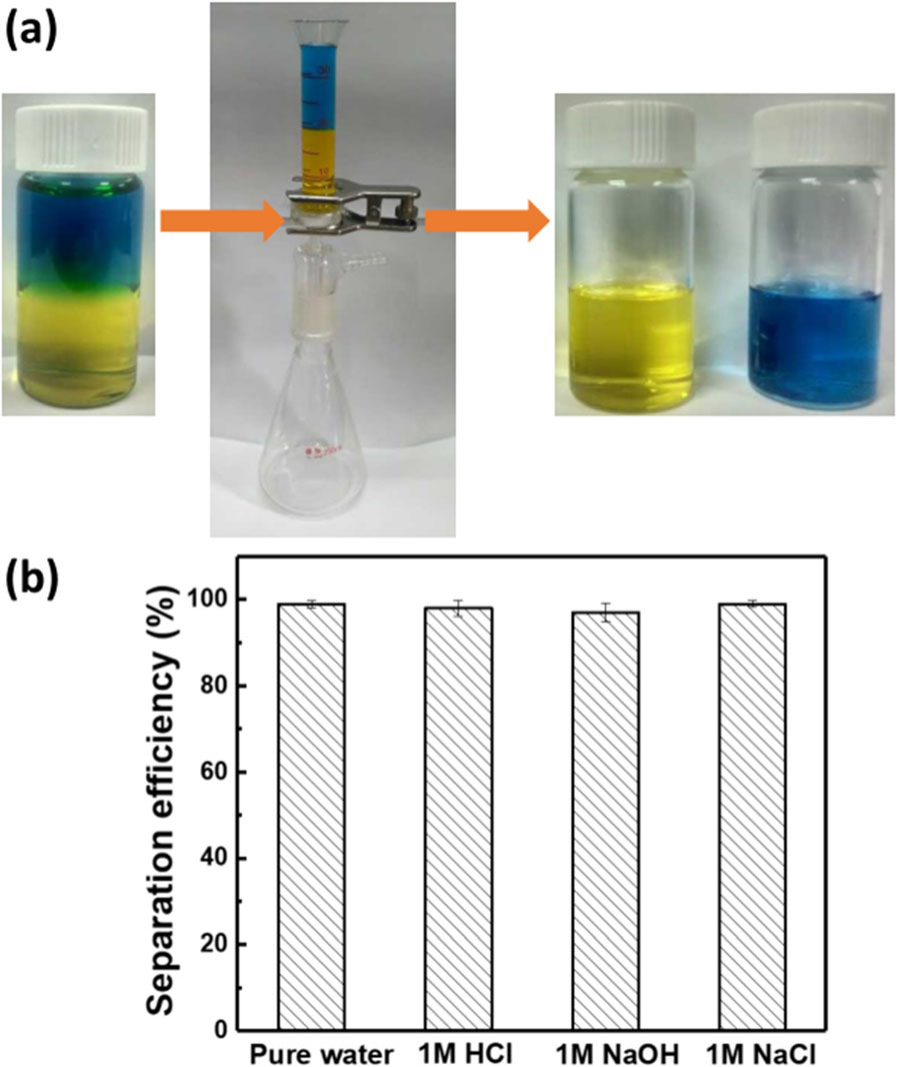
**a** Oil/water separation device and process, **b** separation efficiencies for immiscible oil/water mixtures containing different water phase by the modified F-60 membrane

Gravity driven oil/water separation has been achieved by many hydrophobic or hydrophilic membranes contained one-dimensional components [37–40]. Therefore, the modified F-60 membrane with hydrophobicity was firstly used for the separation of immiscible oil/water mixtures. The oil phase is carbon tetrachloride and the water phase is pure water, which are stained by methyl red and methylene blue, respectively. The oil/water separation process is carried out in a simple oil/water separating device as shown in Fig. 6a. The modified F-60 membrane was fixed between two glass tubes. When the oil/water mixture is poured onto the membrane, carbon tetrachloride permeated the membrane while water is kept in the upper side. Ten milliliters of carbon tetrachloride can pass through the membrane in 240 s. The calculated membrane flux is about 849 L m^−2^ h^−1^ and the separation efficiency for immiscible oil/water mixtures by the modified F-60 membrane reaches up to 99.7%. Generally, the water phase is not neutral especially for oily industrial wastewater. Figure 6b indicates that the modified F-60 membrane keeps high separation efficiency and even water phase contains corrosive acid, alkali, or salt.

**Fig. 7:**
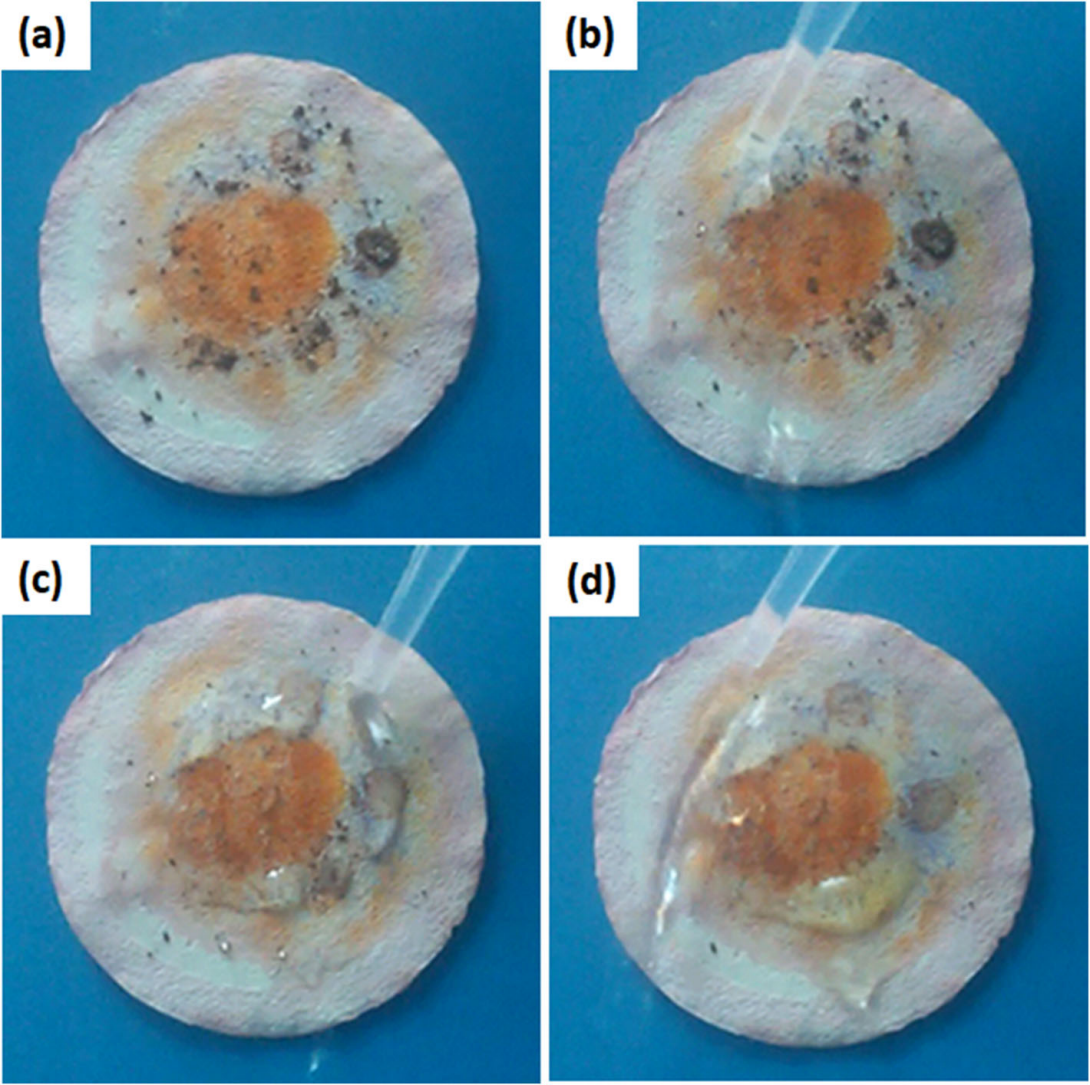
Digital images of self-cleaning process

Except for the different chemical contents in water, there is always dust or solid in industrial wastewater. Figure 7 indicates that the dust staying on the membrane after oil/water separation can be easily removed by water droplets due to the hydrophobic surfaces of the modified F-60 membrane.

**Fig. 8:**
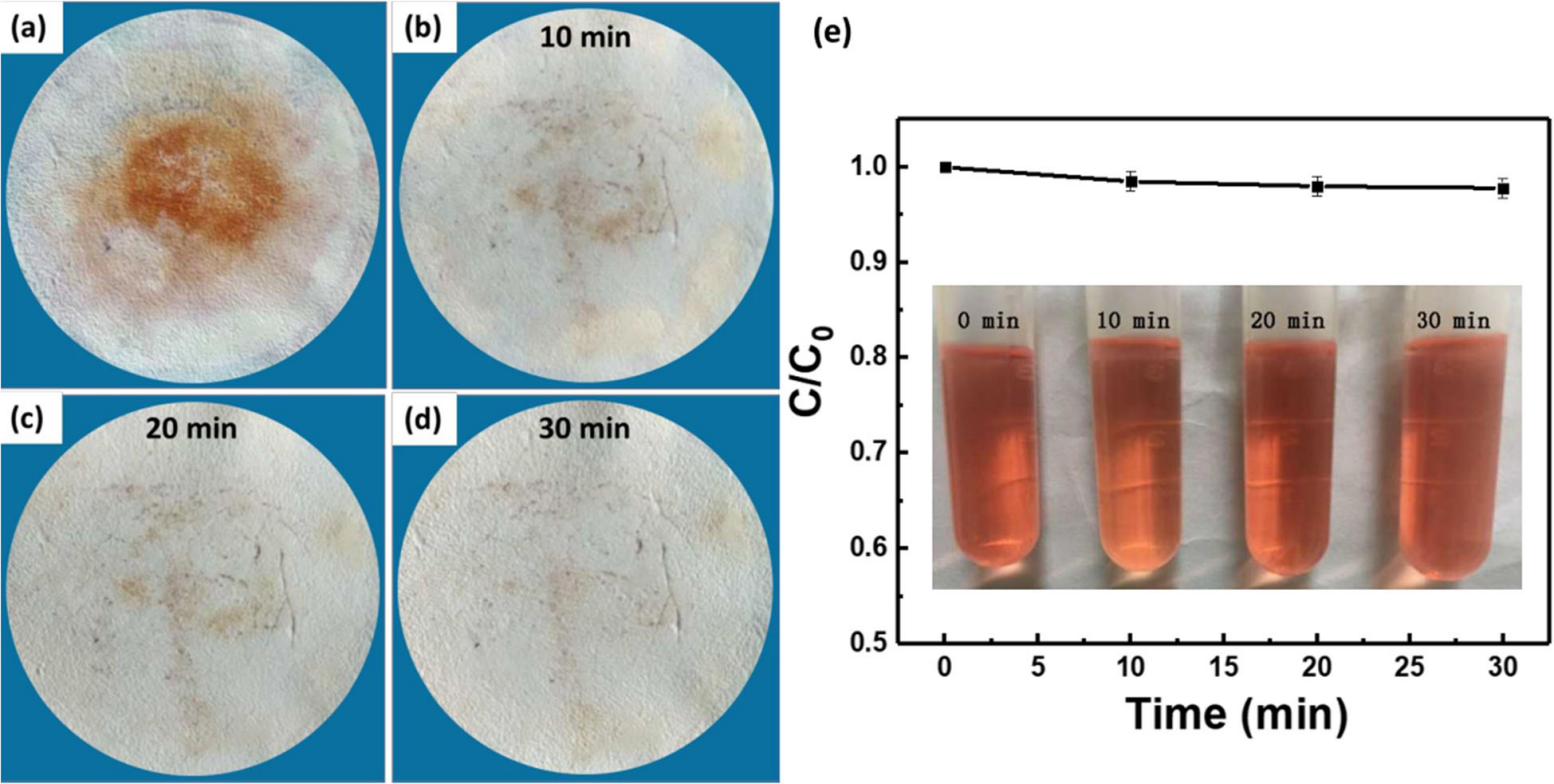
Optical picture of the membrane after oil/water separation and self-cleaning (**a**) and the optical pictures of this membrane irradiated under UV-light for 10 min (**b**), 20 min (**c**), and 30 min (**d**). **e** Degradation efficiency of methyl red solution without photocatalyst under irradiation of UV-light. Inset is the optical photo of methyl red solution irradiated at different times

The properties of materials contained in the membrane usually endow the membrane some special functions [41–43]. The membrane prepared using cross-linked cardanol-graphene oxide contains not only oil/water separation function but also a marked antibacterial activity that originates from the cardanol [44]. Here, the specific surface areas and average pore diameter of the F-60 membrane are 240.4 m^2^ g^−1^ and 14.5 nm, respectively (Additional file 1: Figure S4). This porous structure and high specific surface area of the membrane may have a high adsorption capacity. Figure 7 indicates that after the oil/water separation process, the dye of methyl red in the oil phase can be partly adsorbed on the membrane. The self-cleaning process cannot clean the adsorbed dye. Taking advantage of photocatalytic property of sodium titanate [45–47], the adsorbed dye is expected to remove through photocatalysis reaction. Figure 8a–d displays that after 30 min irradiation with UV-light, almost all the adsorbed dye is removed. In order to demonstrate the removal of methyl red on the membrane due to photocatalysis reaction but not the decomposing of dye under UV-light irradiation, methyl red solution without photocatalyst was irradiated with UV-light. It can be seen from Fig. 8e that without photocatalyst, methyl red cannot be degraded by UV-light, which confirms the photocatalytic function of the sodium titanate membrane.

**Fig. 9:**
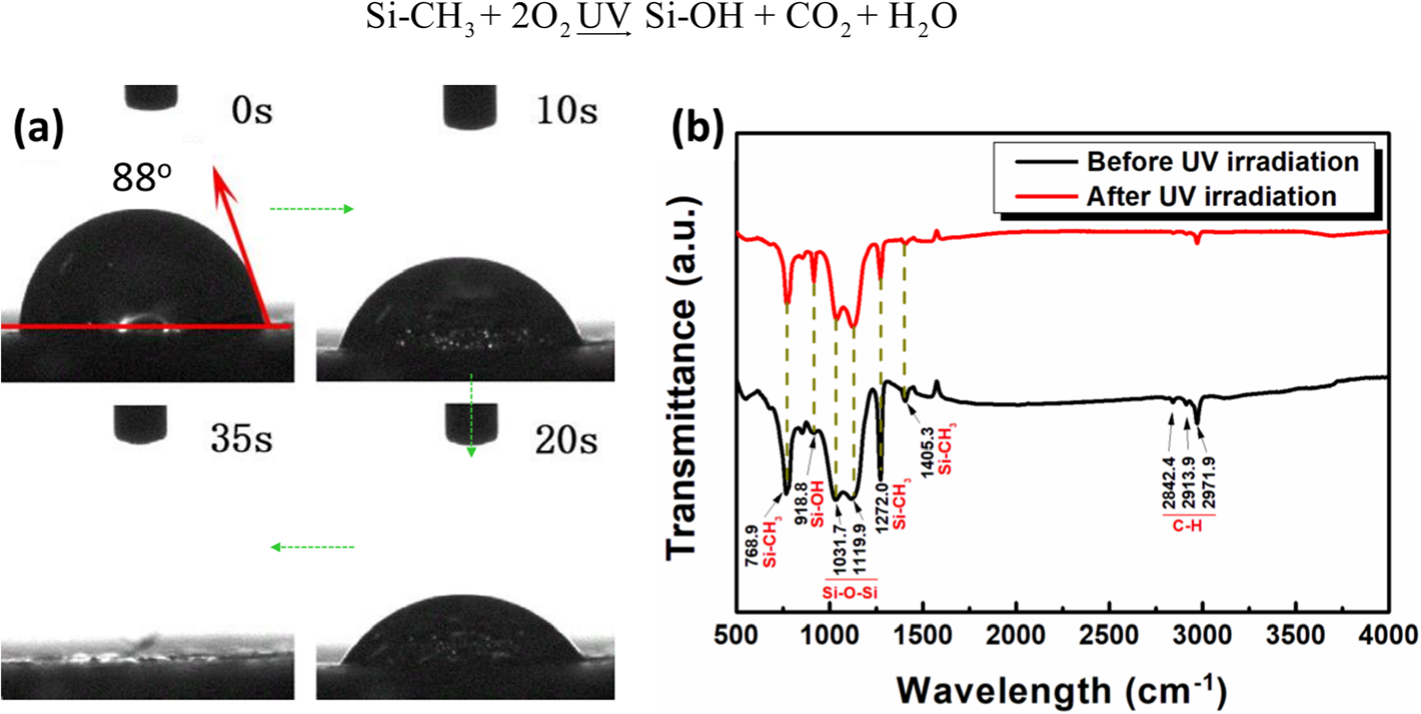
**a** Contact angle of the membrane after UV-light irradiation and **b** FTIR spectra of pure membrane

The MTMS modified F-60 membrane has light transmittance [48], so the Na_2_Ti_3_O_7_ nanotube can adsorb UV-light and generates electrons and holes. But the generation of hydroxyl radicals (Additional file 1: Figure S5) and the degradation of organic molecules need the medium of water. For investigating the mechanism of photocatalytic degradation of the organic molecule by MTMS modified F-60 membrane with superhydrophobic surface, a pure MTMS-modified F-60 membrane was irradiated under UV light for 30 min. It is found that after the irradiation of UV light, the contact angle of the membrane sharply decreased from 150.4° to less than 90° (Fig. 9a). This means that the surface property of MTMS-modified F-60 membrane changes. The FTIR result confirms that after UV light irradiation, Si–O–Si bonds in MTMS decrease, indicating these bonds are broken by UV light (Fig. 9b) [49–52]. The broken Si–O–Si will help the contact of water and light with Na_2_Ti_3_O_7_ nanotube and enhancing the photocatalytic performance. Furthermore, under the combined action of UV light and oxygen, MTMS is oxidized and more Si–OH bonds are observed in Fig. 9b; the reaction is shown in Eq. (1):

1 $$ \mathrm{Si}-{\mathrm{CH}}_3+{2\mathrm{O}}_2\underrightarrow{\mathrm{UV}}\ \mathrm{Si}-\mathrm{OH}+{\mathrm{CO}}_2+{\mathrm{H}}_2\mathrm{O} $$

The broken Si–O–Si and oxidization of Si–CH_3_ by UV light make the generation of hydroxyl radicals and the degradation of organic molecular possible. When this membrane, after irradiation under UV light, was re-dipped in MTMS sol for a very short time, the contact angle of the membrane can rise back to 140° (Additional file 1: Figure S6). The recovery membrane can be reused for immiscible oil/water mixture separation and still preserve self-cleaning and photocatalysis functions. Currently, the membrane only can be recycled three times because the continuous increase of MTMS thickness results in a dramatic decrease of porosity of the membrane (Additional file 1: Figure S7). Studies are still ongoing for further improvement of the recovery rate of the membrane.

The above results indicate that the sodium titanate membrane preserves the multifunction of oil/water separation, self-cleaning, and photocatalysis simultaneously. Inorganic materials endow membranes multifunctional, which are required for treating industrial wastewater (Table 2).

**Table 2:** Representative summary of oil/water separation membranes incorporating 1D inorganic materials

	Components	Water permeability	Other functions	Ref
Pressure-induced separation	SiO_2_ nanofibers and nanostructured MnO_2_	4.9 × 10^5^ L m^−2^ h^−1^ (under the pressure of 5 kPa)	Catalytic activity	[15]
	Sodium titanate nanofibers and cellulose microfibers	6.8 × 10^4^ L m^−2^ h^−1^ bar^−1^	Antifouling property	[22]
	Single-walled carbon nanotube and TiO_2_	3 × 10^4^ L m^−2^ h^−1^ bar^−1^	Antifouling property Self-cleaning	[41]
	TiO_2_ nanofibers and Ag nanoparticles	~ 4.5 L m^−2^ h^−1^ bar^−1^	Photocatalysis Antibacterial activity	[16]
Gravity-triggered separation	Silica nanofibrous and NiFe_2_O_4_ nanoparticles	1580 L m^−2^ h^−1^	Magnetic responsiveness Dye adsorption	[42]
	Fluorinated silica nanofibrous/Al_2_O_3_	892 L m^−2^ h^−1^	Antifouling property	[40]
	TiO_2_ nanofibers and rGO sheets	–	Photocatalysis	[43]
	Sodium titanate ultralong nanotubes	849 L m^−2^ h^−1^	Self-cleaning Photocatalysis	This work

## Conclusions

In summary, we successfully prepared a multifunctional free-standing membrane with Na_2_Ti_3_O_7_ ultralong nanotubes. The diameter and the length of Na_2_Ti_3_O_7_ ultralong nanotubes are about 48 nm and hundreds of micrometers, respectively. The elongated Na_2_Ti_3_O_7_ ultralong nanotubes are easy to lay flat to form a membrane. The contact angle of the membrane can reach up to 150.4° after modifying by MTMS. The MTMS-modified free-standing membrane exhibits high membrane flux of 849 L m^−2^ h^−1^and separation efficiency of 99.7% for immiscible oil/water mixtures, even in strong alkaline, acid, or corrosive salt conditions. Additionally, the residual dust can be removed by self-cleaning function and adsorbed dyes on the membrane can be degraded in 30 min by photocatalytic function of the membrane. The free-standing sodium titanate membrane with a variety of functionalities of oil/water separation, self-cleaning, and photocatalysis will promise wide applications in environmental remediation and wastewater purification.

## Additional File

**Additional file 1: Figure S1.** (a) XPS spectrum of Na_2_Ti_3_O_7_ and (b) atomic ratios of elements calculated from XPS spectrum. **Figure S2.** SEM images of section thicknesses of films (a) F-30, (b) F-45. (c) F-60, (d) F-75. **Figure S3.** SEM images of modified F-60 film with MTMS aged for 14 h. **Figure S4.** (a) Nitrogen adsorption-desorption isotherm and (b) pore size distribution of F-60 film. **Figure S5.** Radical trapping experiments. **Figure S6.** Contact angle of the membrane after recovery. **Figure S7.** SEM image of F-60 membrane after the fourth time modified by MTMS.

## Data Availability

The datasets used and/or analyzed during the current study are available from the corresponding author on reasonable request.

## Abbreviations

CA: Contact angle
F-30, F-45, F-60, and F-75: Membranes with weights of 30 mg, 45 mg, 60 mg and 75 mg, respectively
HRTEM: High-resolution transmission electron microscope
MTMS: Methyltrimethoxysilane
P25: TiO_2_ powder
SEM: Scanning electron microscope
TEM: Transmission electron microscope
UV: Ultraviolet
XRD: X-ray diffraction
