# Supplementary material for: Free-Standing Sodium Titanate Ultralong Nanotube Membrane with Oil-Water Separation, Self-Cleaning, and Photocatalysis Properties
Source: Nanoscale Res Lett. 2020 Jan 28;15:22. doi: 10.1186/s11671-020-3255-9 (PMC6987293; doi:10.1186/s11671-020-3255-9)
Supplement: Supplementary file 1 — Additional file 1: Figure S1. (a) XPS spectrum of Na2Ti3O7 and (b) atomic ratios of elements calculated from XPS spectrum. Figure S2. SEM images of section thicknesses of films (a) F-30, (b) F-45. (c) F-60, (d) F-75. Figure S3. SEM images of modified F-60 film with MTMS aged for 14 h. Figure S4. (a) Nitrogen adsorption-desorption isotherm and (b) pore size distribution of F-60 film. Figure S5. Radical trapping experiments. Figure S6. Contact angle of the membrane after recovery. Figure S7. SEM image of F-60 membrane after the fourth time modified by MTMS. [file 11671_2020_3255_MOESM1_ESM.docx]

**Free-Standing Sodium Titanate Ultralong Nanotubes Membrane with Oil-Water Separation, Self-Cleaning and Photocatalysis Properties**

**Shuling Shen*, Cheng Wang, Minquan Sun, Mengmeng Jia, Zhihong Tang, Junhe Yang***

School of Materials Science and Engineering, University of Shanghai for Science and Technology, Shanghai, 200093, P. R. China.

Email: slshen@usst.edu.cn; jhyang@usst.edu.cn


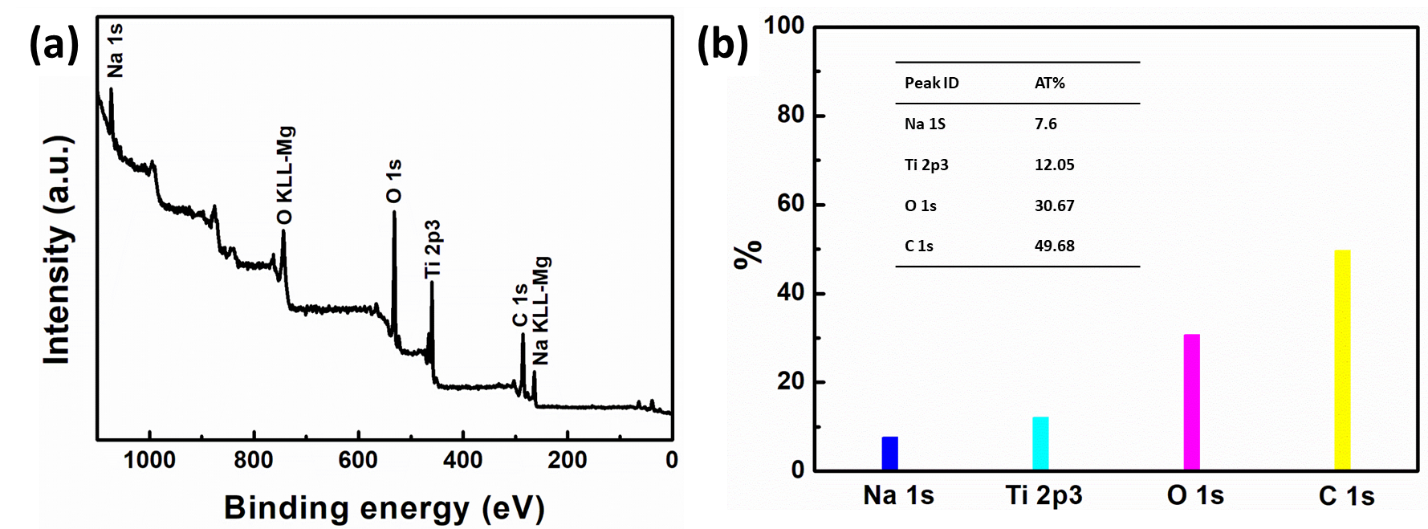


**Figure S1** (a) XPS spectrum of Na_2_Ti_3_O_7_ and (b) atomic ratios of elements calculated from XPS spectrum.


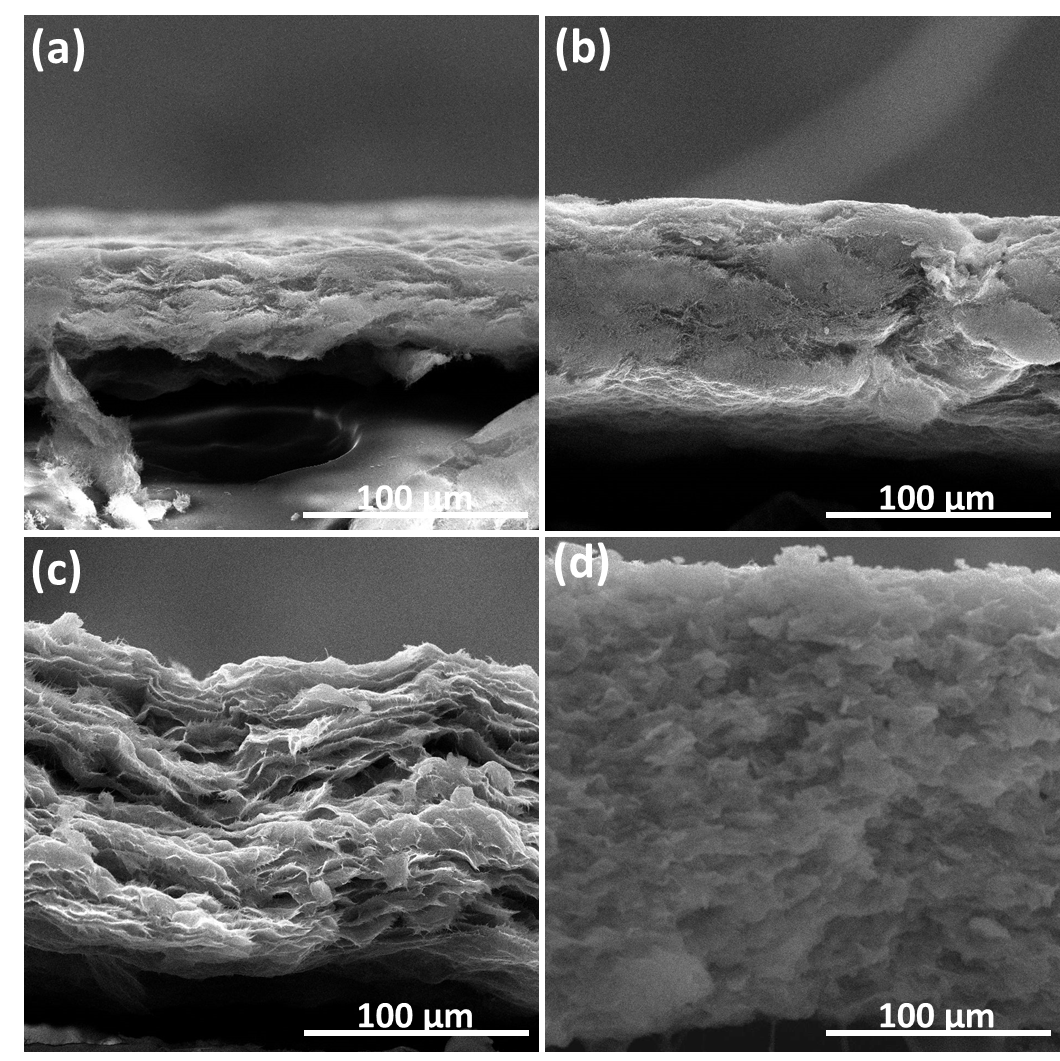


**Figure S2** SEM images of section thicknesses of films (a) F-30, (b) F-45. (c) F-60, (d) F-75.


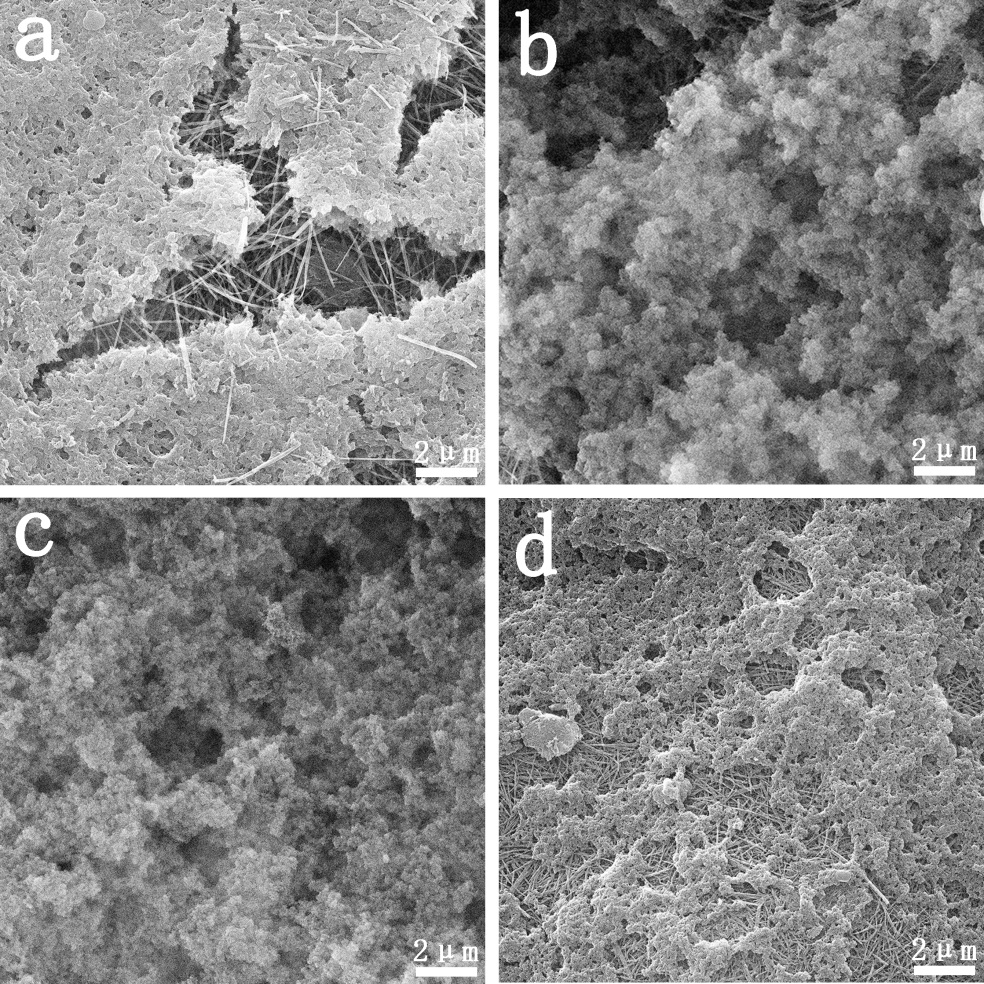


**Figure S3** SEM images of modified F-60 film with MTMS aged for 14 h.


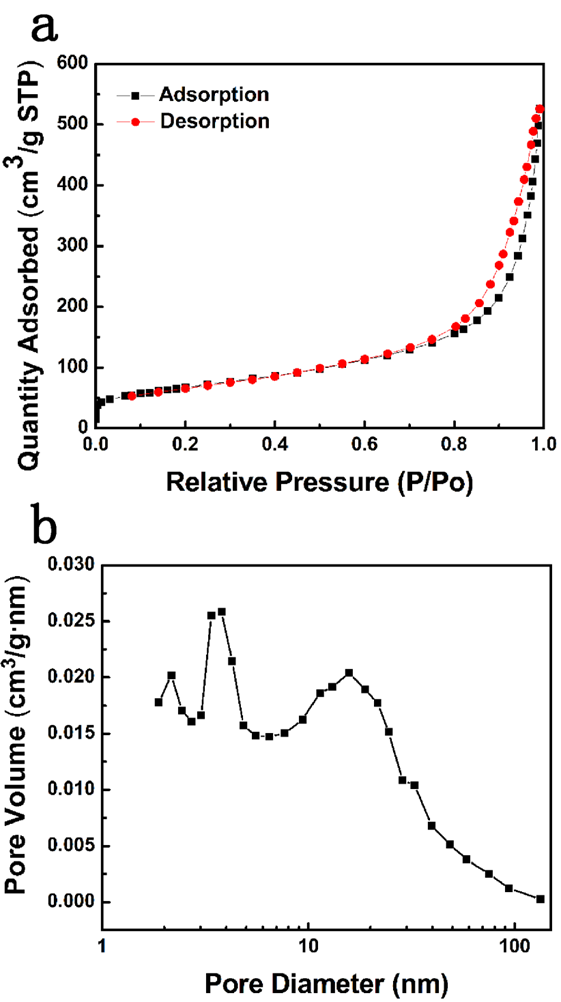


**Figure S4** (a) Nitrogen adsorption-desorption isotherm and (b) pore size distribution of F-60 film.


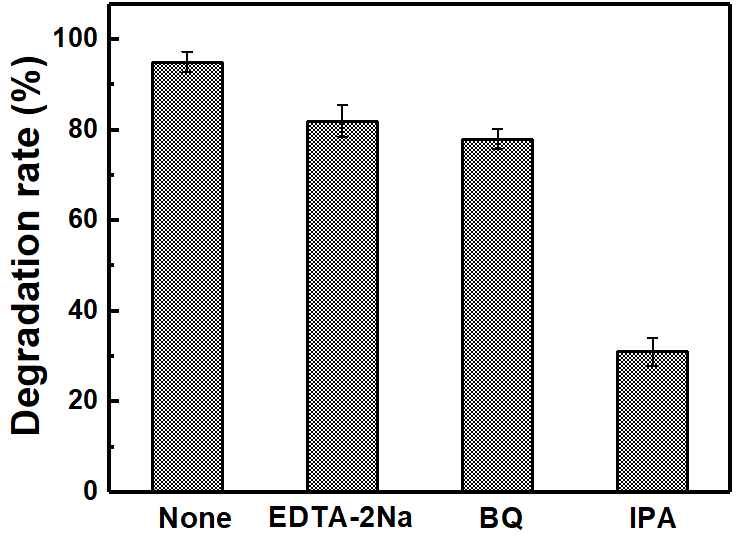


**Figure S5** Radical trapping experiments.

To determine the radicals involved in the photocatalytic reaction and further understand the photocatalysis mechanism over the MTMS modified F-60 membrane, radical trapping experiments were carried out. The ethylenediaminetetraacetic acid disodium salt (EDTA-2Na, 2 mmol/L), 1,4-benzoquinone (BQ, 0.2 mmol/L) and isopropyl alcohol (IPA, 2 mmol/L), were respectively used to scavenge the h^+^, ·O^2−^, and ·OH species during the photocatalytic processes. The corresponding degradation rate of methyl red molecular are shown in Figure S5. The result revealed that the photocatalytic degradation of methyl red over the MTMS modified F-60 membrane decreased slightly in the presence of EDTA-2Na and BQ, indicating that h^+^ and ·O^2−^ radicals had a slight influence on the photocatalytic performance of the MTMS modified F-60 membrane. The photocatalytic activity of the MTMS modified F-60 membrane was obviously decreased by adding IPA. It suggests that ·OH radicals play an important role in the methyl red degradation.


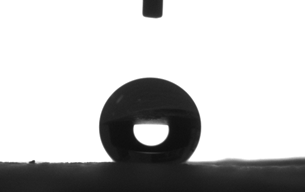
**Figure S6** Contact angle of the membrane after recovery.


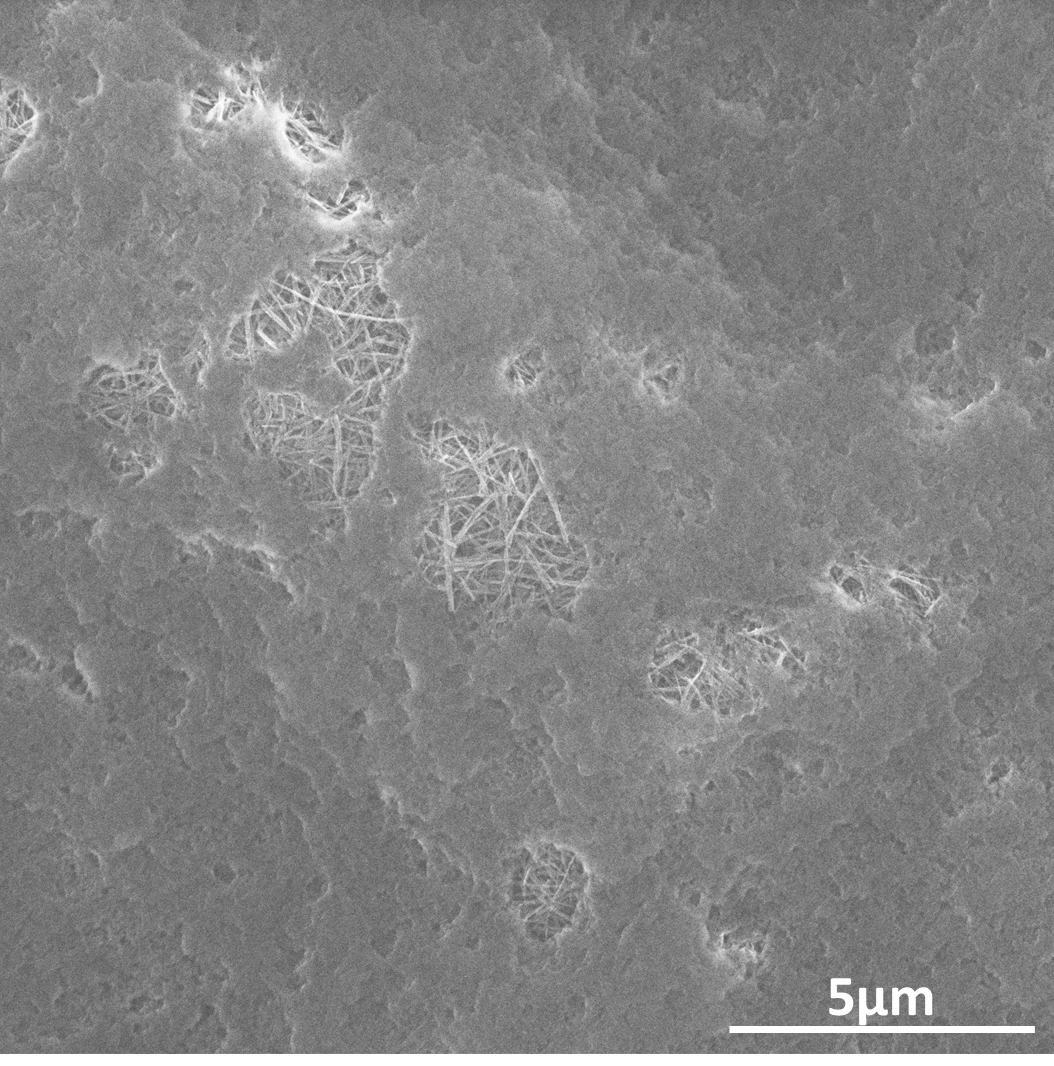


**Figure S7** SEM image of F-60 membrane after the fourth time modified by MTMS.
